# Longitudinal surveillance of group A streptococcal pharyngitis and impetigo in remote Western Australian school children informs acute rheumatic fever prevention

**DOI:** 10.1371/journal.pgph.0005398

**Published:** 2025-12-19

**Authors:** Janessa Pickering, Dylan D. Barth, Bernadette Wong, Elizabeth McKinnon, Marianne Mullane, Alexandra Whelan, August Mikucki, Rebecca Dalton, Abbey Ford, Gelsa Cinanni, John Joseph, Andrew J. Hayes, Mark R. Davies, Alana Whitcombe, Nicole J. Moreland, Liam Bedford, Scott Winslow, John Jacky, Robyn Macarthur, Shelley Kneebone, Narelle Ozies, Delia Lawford, Jonathan Carapetis, Asha C. Bowen, on Behalf of the Missing Piece Study Surveillance Team

**Affiliations:** 1 Wesfarmers Centre for Vaccines and Infectious Diseases, The Kids Research Institute Australia, University of Western Australia, Perth, Western Australia, Australia; 2 School of Biomedical Sciences, University of Western Australia, Perth, Western Australia, Australia; 3 Institute for Health Research, University of Notre Dame, Fremantle, Western Australia, Australia; 4 PathWest Laboratory Medicine, Perth, Australia; 5 Department of Microbiology and Immunology, The University of Melbourne at the Peter Doherty Institute for Infection and Immunity, Victoria, Australia; 6 The Maurice Wilkins Centre for Molecular Biodiscovery, The University of Auckland, Auckland, New Zealand; 7 School of Medical Sciences, Faculty of Medical and Health Sciences, The University of Auckland, Auckland, New Zealand; 8 Derby Aboriginal Health Service, Derby, Western Australia, Australia; 9 Broome Regional Aboriginal Medical Service, Broome, Western Australia, Australia; 10 Department of Infectious Diseases, Perth Children’s Hospital, Nedlands, Western Australia, Australia; 11 Menzies School of Health Research, Charles Darwin University, Darwin, Northern Territory, Australia; 12 Institute of Perioperative Excellence, School of Medicine, University of Western, Perth, Western Australia, Australia; University of Colorado Anschutz Medical Campus: University of Colorado - Anschutz Medical Campus, UNITED STATES OF AMERICA

## Abstract

The prevalence of impetigo and pharyngitis – which are both superficial group A streptococcus (GAS) infections that precede acute rheumatic fever – is poorly defined. Guidelines recommend the early diagnosis of both infections to prevent ARF; however, screening to enable the concurrent detection of these infections in high-risk populations has rarely been performed. In this observational cohort study, children attending one of two schools in the remote Kimberley region of Western Australia were assessed for signs and symptoms of impetigo and pharyngitis at repeated screening visits (conducted up to three times per year), and weekly assessments were performed in response to self-reported symptoms. Throat and skin swabs and dried blood spots were collected at screens. Swabs underwent standard microbiological culture and whole genome sequencing was conducted on confirmed GAS isolates. Dried blood spots were assessed for anti-streptococcal antibody titres. A higher-than-anticipated rate of pharyngitis (29.5%), GAS-positive pharyngitis (6.3%) and GAS carriage (9%) was detected, but GAS-positive impetigo was lower (2.6%) compared with previous studies in the Kimberley. Aboriginal and Torres Strait Islander children experienced more GAS infections than did children of other ethnicities, whereas anti-streptococcal antibody titres did not differ according to ethnicity. This study provides evidence to support the need for increased investment and resourcing of ARF primary prevention in the Kimberley, due to high rates of GAS infection.

## Introduction

Acute rheumatic fever (ARF) – a serious consequence of a group A *Streptococcus* (GAS) infection – can cause permanent damage to heart valves leading to rheumatic heart disease (RHD) [1]. RHD results in heart failure, stroke and death, and is the leading cause of non-congenital cardiovascular deaths in children and adolescents. ARF and RHD are inextricably linked to poverty and are most prevalent in low- and middle-income settings, and within Indigenous communities in high-income countries such as Australia [2].

Australians living in the Kimberley region located in the north of the state of Western Australia experience high levels of socioeconomic disadvantage; this is associated with health inequalities compared to the broader Australian population. The Kimberley region encompasses a vast area of 421,000 square kilometres (~three times the size of the England) and is home to ~35,000 people, 47% of whom are Aboriginal. The population is dispersed in six major towns and over 200 small and remote communities, many of which have widespread poor living conditions that complicate efforts to prevent GAS infections. The most recent ARF prevalence estimates for Aboriginal people in the Kimberley are 183/100,000 people below 45 years, the second highest regional prevalence in the country and among the highest reported rates globally [2].

Pharyngitis caused by GAS has long been considered necessary for the development of ARF [3,4]. However, recent studies have shown that impetigo and throat carriage represent important additional pathways to ARF. This includes a large data-linkage study from New Zealand showing that GAS-positive impetigo independently increases the risk of first ARF hospital admission [5]. Additionally, contemporary Australian studies have highlighted high prevalences of GAS-positive impetigo in populations with high rates of ARF [6,7]. Recent studies in Australia [8] and The Gambia [9] have identified pharyngeal GAS carriage as a driver of GAS transmission amongst households, for both subsequent skin and throat infections. Historically, northern Australia has provided conflicting evidence on the prevalence of GAS-positive pharyngitis in populations at risk of ARF, with both low and high rates reported across all ages [10–12]. By conducting the Missing Piece Study, we sought to understand the concurrent burden of GAS throat and skin infections in a population of children living in a geographical region placing them at high risk of developing ARF.

Our study aims were to determine the epidemiology of GAS-positive pharyngitis, pharyngeal GAS carriage, and GAS-positive impetigo among children in the remote Kimberley region of Western Australia through school-based prospective surveillance. We report a component of the Missing Piece Study pertaining to all GAS infections in the cohort, particularly the prevalence determined by culture, insight into the performance of validated clinical diagnostic rules, molecular epidemiology of GAS strains and *streptococcal* serology at time of GAS isolation.

## Methods

The Missing Piece Study is a multicomponent study in which data were collected from the prospective surveillance of school children, aged 5–15 years, living in a remote region of Western Australia. The published Missing Piece Study protocol [13] described the study’s overarching objectives and detailed the various stand-alone and nested studies that it comprised.

### Ethics statement

This study was endorsed by the Kimberley Aboriginal Health Planning Forum (Ref 2018:016) before subsequent approvals were sought. Ethical approval for the complete study protocol and component analyses were obtained from the Western Australian Aboriginal Health Ethics Committee (Ref: 892), the Human Research Ethics Committee of the University of Western Australia (Ref: RA/4/20/5101) and the Catholic Education office of Western Australia. The original study protocol was amended and approved by ethics boards to enable evaluation of point-of-care testing, microbiome analysis of throat swabs and study length due to COVID lockdown interruptions. Parents or guardians provided formal written consent for children to participate in the study and children (participants) gave their assent to participate in a physical assessment and specimen collection at each visit.

### Study setting and study partners

GAS surveillance was conducted in two towns in the Kimberley region of Western Australia (S1 Fig): Broome – which is the largest town in the Kimberley region and has a population of around 14,600 residents – and Derby, which is 225 km north-east of Broome and has a smaller population of around 3,200 residents (Australian Bureau of Statistics: 2021 Census QuickStats). Both the Kimberley Aboriginal Medical Service and the Western Australian Country Health Service provide and manage health clinics in the region; these are clinically staffed by nurses, general practitioners, and Aboriginal health workers and practitioners. The Broome Regional Aboriginal Medical Service and the Derby Aboriginal Health Service were integral study partners who performed and enabled surveillance activities at school sites and managed the treatment of participants referred through the study. Catholic Education Western Australia endorsed and enabled visits to schools under their governance.

### Study design and collection of surveillance data

**Fig 1:**
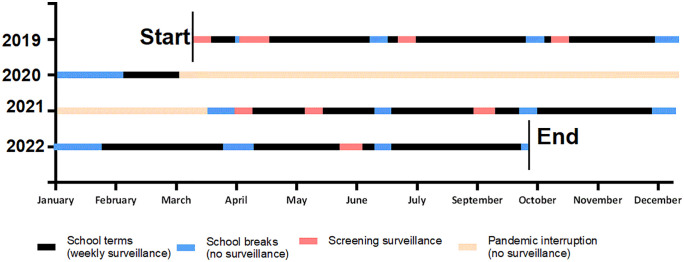
Longitudinal surveillance timeline. Children participating in the study attended visits conducted over 4 school terms (shown in black) that were separated by school holiday breaks (shown in blue). No study visits were conducted during school holidays (blue) or during the pandemic-interruption period (shown in peach). Prospective-screening visits (shown in red) were conducted at each of the 2 participating schools between 1 and 3 times per year.

Details of study design and data collection have been outlined previously [13]. Briefly, the study involved school-based recruitment of participants who attended a single participating school in either Broome or Derby. Both are private schools that accept fees for tuition. Families of all children attending the school were invited to enrol and children were excluded if they were aged below 5 or above 15. Recruitment began on 4^th^ of February 2019 and remained open until the final date of study activity (September 21^st^ 2022). Two types of study visit were conducted: 4–12-monthly prospective-screening visits that were attended by all available participants, regardless of their symptomatology, and weekly active-screening visits that were attended only by participants with self-reported symptoms. Fig 1 depicts the timing of the study visits, which were halted temporarily due to lockdowns and travel restrictions imposed to limit the spread of COVID-19 in the Kimberley between March 2020 and April 2021.

During prospective-screening visits, each participant was invited to complete the following activities: 1) answer questions from a sore-throat checklist for assessment by the study team; 2) undergo a clinical examination of their throat and skin; 3) undergo the collection of throat and skin lesion swabs to detect the presence of GAS; 4) photographs of throat and skin, and 5) provide finger-prick blood samples to detect antibodies to streptococcal antigens.

During active-screening visits, only children who self-reported pharyngitis or impetigo symptoms on the day of the visit completed activities 1–5 as described for the prospective-screening visits. Development of the self-report sore throat checklist is previously described [14]. Pharyngitis was defined as any of: having a self-reported sore throat; having more than 2 self-reported symptoms that are indicative of a sore throat, such as finding it hard to swallow and subsequently avoiding eating or drinking; or having at least 2 signs of pharyngitis: fever, enlarged tonsils, tonsillar erythema, tonsillar exudate, or tender or enlarged lymph nodes. Pharyngitis cases were further classified as GAS positive or GAS negative according to results from the microbiological culturing of throat swabs. Pharyngeal GAS carriage was defined as a participant without pharyngitis having a GAS-positive culture from a throat swab.

Active impetigo was defined as described previously [15] comprising either a purulent or crusted sore. Active impetigo was further classified as either GAS positive or negative following microbiological culturing of skin sore swabs.

### Handling and processing of biological specimens

Throat and skin swabs (Copan, Italy) were collected using processes described previously [13,15,16] then stored at 4–8°C in tubes containing skim milk glucose glycerol broth (PathWest Media, Perth, Australia). Each swab was transported to Perth via aeroplane by a medical courier while being kept at 4–8°C. On arrival at The Kids Research Institute laboratories, Perth, Australia, each swab tube was thoroughly vortexed then stored at –80°C. Finger-prick blood specimens were collected onto Whatman cards (Sigma Aldrich, Merck Life Science Pty Ltd, Bayswater, Victoria, Australia), air-dried overnight, and stored individually within foil seals containing desiccant to keep them dry during transport and long-term storage at –80°C.

Microbial culturing was performed as described previously [13]. One group A isolate per swab was cultured again onto horse blood agar overnight for genomic DNA extraction. A single colony was inoculated into 1.2 mL Todd–Hewitt Broth (Bacto Laboratories, NSW, Australia) with one percent yeast extract and grown overnight at 37°C. Overnight cultures were pelleted via centrifugation and resuspended in lysin buffer A for lysis by B30 phage lysin as previously described [17]. DNA was extracted from lysates using the Qiagen DNeasy Blood and Tissue Kit and eluted into nuclease-free water following manufacturer’s instructions. DNA quality and concentration were assessed using the Qubit Fluorometer (Thermofisher Scientific, Malaga, Western Australia), the NanoDrop (Thermofisher Scientific), and SYBR Safe (Invitrogen, Thermofisher Scientific) visualisation by 1% agarose gel electrophoresis (Thermofisher Scientific).

### Whole genome sequencing and analysis

Genomic DNA from pure bacterial isolates from swabs collected between 2019 and 2021 were sequenced on the Illumina platform Hi Seq 2500 platform using 75–125 bp paired reads (The Wellcome Trust Sanger Institute, United Kingdom). Genomic DNA from isolates collected during 2022 were sequenced on the Illumina NovaSeq platform using 150 bp paired reads (Australian Genome Reference Facility, Australia). All genomic data were analysed using the Bohra analysis pipeline version 2.3.2 [18]. Briefly, raw reads were filtered for quality and species identity, assembled using shovill v1.1.0 [19], and annotated with prokka v1.14.6 [20]. This pipeline was also used to determine the MLST type (mlst v2.23.0) [21] and *emm* type (emmtyper v0.2.0) [22] of each GAS isolate and to detect markers for antimicrobial resistance (abritamr v1.0.14) [23]. Core genome SNPs were detected with snippy v4.4.5 [24] and a phylogeny generated from the resulting alignment using IQTree v2.1.4-beta [25]. The genome of MGAS5005 (*emm1,* ST28) was used as the reference genome (RefSeq GCF_000011765.3). The phylogenetic data were visualised in R v4.3.1 using the treeio (v1.24.3) [26], ggtree (v3.8.2) [27], and ggtreeExtra (v1.10.0) [28] packages.

### Measuring anti-streptococcal antibody titres

Two methods were used to measure streptococcal antibody titres from dried finger-prick blood spots: nephelometry and bead-based immunoassay. Nephelometry was performed by the PathWest laboratory service provider to measure anti-streptolysin O titres (ASOT) [29]. A bead-based 8-plex immunoassay was performed to measure antibodies against 8 GAS antigens using a Luminex platform [30]. To evaluate the comparability of results determined by either method, a Spearman’s correlation coefficient was calculated (S2 Fig).

### Data handling and statistical analysis

Surveillance data were collected and handled as described previously [13]. Data quality was checked by three personnel and data were cleaned prior to analysis. The prevalences of pharyngitis and impetigo were each calculated as the proportion of culture-confirmed screening assessments performed during the study period that identified a positive case. Prevalence and symptom data were analysed using RStudio (RStudio Team, 2016; www.rstudio.com/). Log-binomial regression with cluster-robust standard errors was used for prevalence analyses. For bead-based immunoassay results, mean anti-streptolysin O titres (IU/mL) were derived by interpolation as described [31]. Mann Whitney test was used for comparisons of antibody titres. P-values are not adjusted for multiple comparisons.

## Results

### Study period and participant demographics

All study visits, including those for prospective-screening and active screening, were performed between March 2019 and September 2022 (Fig 1). A total of 254 participants attended screening visits at least once (Table 1), and School 1 included twice as any many participants compared with School 2. Twenty-eight participants enrolled but did not attend a single screening visit (prospective or active) due to their unavailability (through absence, relocation, or graduation) during prospective screening or a lack of symptoms during active screening. The median participant age at enrolment was 8.45 years (Interquartile Range [IQR] 6.7–10.7 years), and more males (n = 146, 57%) than females (n = 108, 43%) participated at each school. The ethnicities of participants were reported as Aboriginal, Aboriginal and Torres Strait Islander, African, Asian, not-provided (n = 2), or non-Aboriginal Australian (Table 1).

**Table 1:** Participant demographics.

	School 1 (Derby) N = 92	School 2 (Broome) N = 162
Year of enrolment		
2019	60 (65%)	106 (65%)
2021	29 (32%)	50 (31%)
2022	3 (3.3%)	6 (3.7%)
Ethnicity		
Non-Aboriginal^1^	49 (53%)	77 (48%)
Aboriginal^2^	43 (47%)	85 (52%)
Sex		
Male	53 (58%)	93 (57%)
Female	39 (42%)	69 (43%)
Age group at enrolment		
4–8 years	50 (54%)	90 (56%)
9–14 years	42 (46%)	72 (44%)
Enrolment age (median [IQR] years)	8.6 [6.7, 10.0]	8.4 [6.7, 10.9]
Screens per child (median [range])	2 [1–6]	2 [0-5]
Active surveillance participation	48 (52%)	79 (49%)

^1^Non-Aboriginal ethnicity in this table includes Asian, African and no ethnicity reported (n = 2). ^2^Aboriginal ethnicity in this table includes Aboriginal and Torres Strait Islander. N – total participants.

### Prevalence of pharyngitis, pharyngeal GAS carriage, and impetigo at prospective-screening visits

Prospective-screening visits were conducted on six occasions at School 1 and five occasions at School 2. These visits included 620 individual assessments, which involved all but one participant; over 15% of participants underwent more than three prospective-screening assessments. Pharyngitis was indicated in 29.5% of prospective-screening assessments (Table 2), and 11.1–45.3% of participants reported pharyngitis-like symptoms during a screening visit. Throat swabs were collected during 92% (569/620) of the prospective-screening assessments, 15.3% of which were culture positive for GAS. The estimated period prevalence of GAS pharyngitis and that of pharyngeal GAS carriage are shown in Table 2. The median point prevalence of GAS pharyngitis across the prospective-screening visits was 4.9% (IQR 2.1% to 11.0%).

**Table 2:** Prevalence estimates for pharyngitis, impetigo and GAS positivity from prospective screening assessments.

Participant subgroups	Pharyngitis^a^		GAS-positive throat swab		GAS pharyngitis		GAS carriage^b^		Active impetigo^c^		GAS + ve impetigo	
	%	95% CI	%	95% CI	%	95% CI	%	95% CI	%	95% CI	%	95% CI
**All participants**	29.5	[22.4,38.9]	15.3	[11.7,19.9]	6.3	[3.8,10.6]	9.0	[7.5,10.7]	15.0	[10.3,21.9]	2.6	[1.6,4.2]
**School**												
School 1	34.5	[27.1,43.9]	19.8	[14.6,26.9]	8.3	[4.4,15.8]	11.5	[9.6,13.8]	14.3	[9.8,20.8]	3.4	[1.5,7.5]
School 2	26.4	[17.5,40.0]	12.5	[9.6,16.3]	5.1	[2.6,10.1]	7.4	[6.3, 8.6]	15.4	[8.9,26.7]	2.1	[1.3,3.5]
**Ethnicity**												
Aboriginal	34.1	[26.0,44.6]	18.8	[13.7,25.7]	7.7	[4.5,13.3]	11.1	[8.3,14.7]	19.2	[12.9,28.6]	3.7	[2.1,6.5]
Non-Aboriginal	24.6	[17.2,35.2]	11.4	[7.8,16.7]	4.8	[2.1,11.0]	6.6	[4.9, 8.9]	10.4	[7.0,15.7]	1.3	[0.6,3.2]
**Sex**												
Male	25.5	[19.2,34.0]	15.9	[11.2,22.4]	6.1	[3.7, 9.8]	9.8	[7.1,13.5]	17.7	[12.3,25.4]	3.3	[1.8,6.0]
Female	35.3	[26.1,47.8]	14.4	[10.5,19.8]	6.8	[3.6,12.5]	7.7	[4.7,12.4]	11.1	[6.6,18.6]	1.6	[0.7,3.5]
**Age at enrolment**												
4–8 years	28.1	[20.0,39.5]	15.5	[12.0,20.0]	6.6	[3.6,11.8]	9.0	[7.4,10.9]	15.6	[11.3,21.7]	3.4	[2.1,5.7]
9–14 years	31.0	[23.4,41.0]	15.1	[10.9,20.8]	6.1	[3.7,10.0]	9.0	[6.0,13.4]	14.3	[8.8,23.2]	1.7	[0.6,4.4]
**Screening Era**												
Pre-COVID disruption	23.9	[13.8,41.5]	16.1	[10.3,25.2]	7.0	[3.2,15.5]	9.1	[7.5,11.0]	19.9	[13.8,28.6]	2.7	[1.7,4.4]
Post-COVID disruption	34.7	[29.5,40.8]	14.5	[11.1,18.8]	5.7	[3.1,10.4]	8.8	[6.5,12.0]	10.5	[7.1,15.6]	2.5	[1.1,5.6]

Data are presented as prevalence (%) and [95% confidence interval] which have been derived from log-binomial regression models with cluster-robust standard errors to account for correlation within visits. ^a^ Pharyngitis defined as: i) a self-reported sore throat or >2 self-reported symptoms of sore throat inclusive of finding it hard to swallow, or ii) ≥2 clinical signs consistent with pharyngitis. ^b^ Pharyngeal carriage defined as a GAS positive culture obtained from a throat swab of an asymptomatic participant or participant with symptoms that did not reach criteria as pharyngitis. ^c^ Active impetigo defined as either purulent or crusted skin sores. The pre- and post- COVID-19 disruption eras were April 2019-March 2020 and April 2021-October 2022, respectively pictured in Fig 1.

Active impetigo (purulent or crusted sores) was detected in 15% of prospective-screening assessments; swab specimens were collected from all cases, and 16/93 (17%) grew GAS on microbiological culture. Individual point prevalence values for impetigo ranged from 0 to 8.7% with a median value of 2.7% (IQR 0.7%-4.8%); additional prevalence estimates for impetigo are shown in Table 2.

Compared with non-Aboriginal participants, Aboriginal participants were more likely to have GAS-positive throat swabs, and active impetigo – including GAS-positive impetigo (Table 3). Compared with male participants, female participants were more likely to present with pharyngitis but less likely to have active impetigo. Lower impetigo prevalence was observed at visits conducted in the post-COVID-19 era compared with the pre-COVID-19 era (*p*-value = 0.02).

**Table 3:** Prevalence ratios.

Multivariable results	Pharyngitis		GAS + ve throat swab		GAS + ve pharyngitis		Active impetigo		GAS + ve impetigo	
	PR [95%CI]	P	PR [95%CI]	P	PR [95%CI]	P	PR [95%CI]	P	PR [95%CI]	P
Aboriginal (v Non-Aboriginal)	1.35 [1.00,1.83]	0.05	1.76 [1.19,2.60]	0.005	1.68 [0.67,4.23]	0.27	2.03 [1.41,2.91]	<0.001	3.45 [1.20,9.93]	0.02
Female (v Male)	1.28 [1.01,1.63]	0.05	0.84 [0.55,1.28]	0.42	1.07 [0.72,1.59]	0.73	0.60 [0.40,0.92]	0.02	0.43 [0.15,1.26]	0.13
Age 9–14 years (v 4–8 years)	1.03 [0.77,1.38]	0.84	0.98 [0.76,1.27]	0.91	0.89 [0.60,1.33]	0.58	0.89 [0.66,1.20]	0.44	0.46 [0.17,1.27]	0.13
Post-COVID era (v pre-COVID)	1.40 [0.85,2.31]	0.19	0.91 [0.60,1.39]	0.68	0.80 [0.31,2.08]	0.65	0.53 [0.31,0.90]	0.02	0.99 [0.39,2.49]	0.98

Prevalence ratios (PR) are derived from multivariable log-binomial regression models that jointly consider community, ethnicity, sex, age and era. Calculation of the 95% confidence intervals and *p*-values have used cluster-robust standard errors to take account of the within-visit correlations. The pre- and post- COVID-19 disruption eras were April 2019-March 2020 and April 2021-October 2022 respectively, pictured in Fig 1.

### Prevalence of pharyngitis, pharyngeal GAS carriage and impetigo at weekly active-screening visits

**Fig 2:**
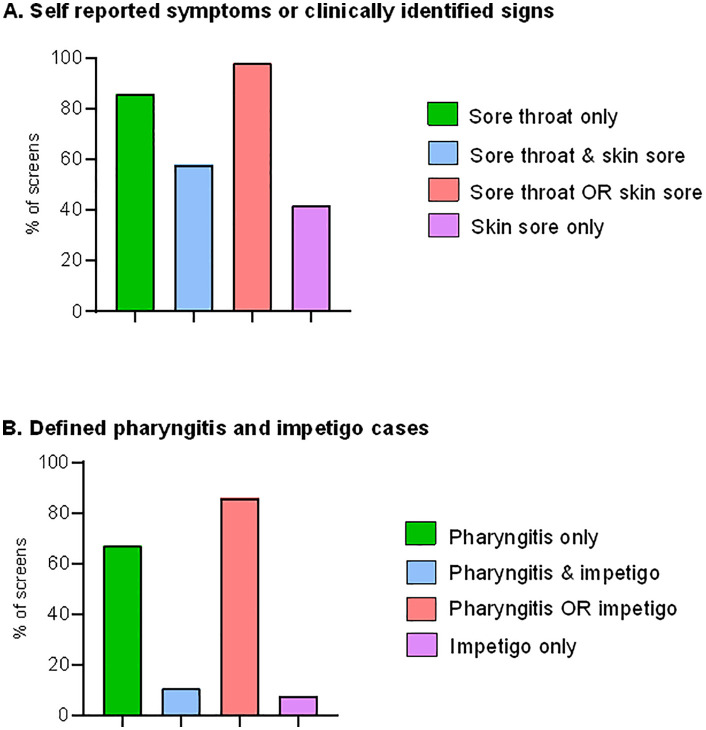
Presentations at weekly active-screening visits. A) all symptomatic throat and skin infections, and B) defined pharyngitis and impetigo during 317 active-screening visits.

A total of 317 active-screening assessments were completed over 116 active-screening visits made during the following periods: weekly School 1 visits took place over 19 weeks during the pre-COVID-19 era (June 2019 to March 2020) and 42 weeks during the post-COVID-19 era (May 2021 to September 2022); weekly School 2 visits took place over 23 weeks during the pre-COVID-19 era (June 2019 to March 2020) and over 32 weeks during the post-COVID-19 era (June 2021 to September 2022). Exactly half of the study participants (127/254) reported symptoms of pharyngitis or impetigo at least once during these periods (at a weekly visit); 31.5% of participants reported symptoms on 1–2 occasions, 13.4% reported symptoms on 3–5 occasions, and 5.1% reported symptoms on 6–10 occasions (Fig 2). There were 20 (6.3%) surveillance visits in which no children reported symptoms.

More than 80% (257/317) of participants who were assessed at an active-screening visit reported at least one symptom of a sore throat, and 77% (244/317) of assessments were consistent with our pharyngitis definition. Active impetigo was confirmed in 15.5% (49/317) of the assessments, with GAS identified in 21.2% (10/47) of the cultures from these skin swabs.

### Symptomatology and diagnostic rules for pharyngitis comparing prospective- and active-screening visits

**Fig 3:**
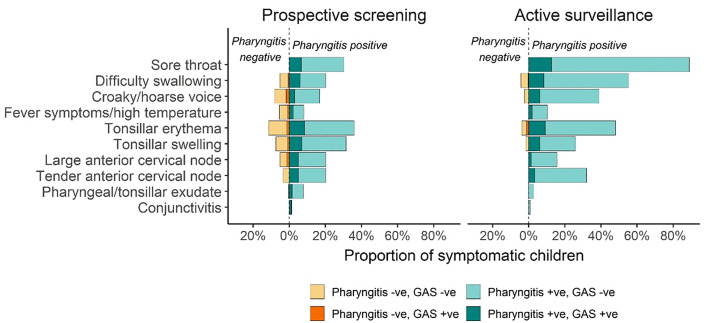
Symptomology of sore throat infections. Denominators are symptomatic children (self-reported and/or identified through physical assessment).

Participants presented with a range of symptoms at the prospective- and active-screening assessments (937 assessments in total); however, the predominant sore-throat symptoms differed according to the type of screening visit (Fig 3, Table 4). During prospective-screening visits, tonsillar erythema (redness) and tonsillar swelling predominated, followed by self-reported sore throat. In contrast, self-reported sore throat was the predominant symptom during active-screening visits (in addition to being a prerequisite for participation in active-screening assessments), followed in descending order by difficulty swallowing and tonsillar erythema. Our pre-determined definition of pharyngitis captured most of the GAS-positive throat swabs among symptomatic participants (Fig 3, proportions in brown text refer to GAS-positive symptomatic individuals not captured by our pharyngitis definition). Across all visit types, the participants that reported a sore throat (n = 333), 94% had at least one additional sign or symptom, 85% had 2 additional signs or symptom and 68% had between 3 and 11 additional signs or symptoms to sore throat pain.

**Table 4:** Symptomology of sore throat infections.

Symptom/clinical sign	Prospective screening				Active surveillance			
	Overall (N = 620)		Throat swab		Overall (N = 317)		Throat swab	
			GAS + ve (N = 87)	GAS -ve (N = 482)			GAS + ve (N = 41)	GAS -ve (N = 251)
	n	(%)	%	%	n	(%)	%	%
Sore throat^s^	92	(14.8)	23.0	14.5	239	(75.4)	80.5	79.7
Difficulty swallowing/eating/drinking^s^	82	(13.2)	23.0	11.4	161	(50.8)	56.1	53.0
Croaky/hoarse voice^s, c^	78	(12.6)	16.1	12.4	112	(35.3)	39.0	36.7
Fever symptoms/high temperature^s, c^	42	(6.8)	11.5	6.2	29	(9.1)	12.2	8.8
Tonsillar erythema^c^	150	(24.2)	33.3	23.2	138	(43.5)	65.9	43.4
Tonsillar swelling^c^	126	(20.3)	27.6	19.1	75	(23.7)	39.0	22.3
Large anterior cervical node^c^	78	(12.6)	21.8	11.6	43	(13.6)	9.8	14.7
Tender anterior cervical node^c^	72	(11.6)	17.2	11.4	88	(27.8)	22.0	29.9
Pharyngeal/tonsillar exudate^c^	25	(4.0)	5.7	3.9	7	(2.2)	0.0	2.8

Notes and abbreviations: n, number of presentations; s: self-reported symptom, c: clinically-identified symptom.

Among the 128 GAS-positive throat swabs collected during this study, 21.1% were collected from children who had no self-reported or physically identified symptoms and so met our definition of pharyngeal GAS carriage; 49% of GAS-positive throat swabs were collected from children who had at least four symptoms, thus meeting our definition of pharyngitis; and 39.8% of GAS-positive throat swabs were collected from children who had between one and three symptoms – participants in this last group were sorted into a ‘pharyngeal carriage’ or ‘pharyngitis’ group based on our definition.

The application of Centor criteria to the 101 symptomatic participants with GAS-positive throat swabs revealed that only 42 (41.6%) of them met the threshold of at least 3 criteria that represents a high probability of GAS-positive pharyngitis; most of these participants (66/101, 65.3%) recorded a Centor score of 2. Among the 101 participants with GAS-positive pharyngitis, fever was rare, with only one child experiencing a temperature >38°C; 89 (88%) of these participants lacked a cough, 23 (22.8%) presented with a tender anterior cervical node and/or lymphadenopathy, and 40 (31.5%) presented with swelling of tonsils or exudate on the pharynx or tonsil.

### Symptomology of impetigo during prospective- and active-screening visits

Almost twice as many skin assessments were performed during prospective-screening visits compared with active-screening visits (165 vs. 89 assessments, respectively, Table 5). In both visit types, the majority of sores were reported to have been present for >5 days. Furthermore, many participants reported that skin sores were itchy and painful. Impetigo swabs that were collected from participants with a GAS-positive throat swab were more likely to be GAS positive than GAS negative.

**Table 5:** Characteristic symptomology in children presenting with evidence of impetigo.

	Prospective screening			Active surveillance		
	Overall (N = 165)	Skin swab		Overall (N = 89)	Skin swab	
		GAS + ve (N = 16)	GAS -ve (N = 149)		GAS + ve (N = 11)	GAS -ve (N = 75)
	n (%)	%	%	n (%)	%	%
Active impetigo	93 (56.4)	100.0	51.7	49 (55.1)	90.9	49.3
Itchy sores	43 (29.3)	15.4	30.6	32 (41.0)	33.3	40.9
Painful sores	42 (28.6)	53.8	26.1	43 (54.4)	55.6	55.2
Duration of sores						
<=3 days	35 (25.7)	38.5	24.4	22 (31.4)	14.3	33.3
4–5 days	18 (13.2)	0.0	14.6	7 (10.0)	0.0	11.7
> 5 days	83 (61.0)	61.5	61.0	41 (58.6)	85.7	55.0
GAS + ve throat swab	30 (19.1)	43.8	16.3	13 (18.3)	50.0	15.0
	**Mean (range)**	**Mean (range)**	**Mean (range)**	**Mean (range)**	**Mean (range)**	**Mean (range)**
Number active sores	1 (0,18)	4 (1,18)	1 (0,10)	1 (0,14)	2 (0,4)	1 (0,14)
Number flat, dry sores	2 (0,20)	3 (0,20)	2 (0,15)	1 (0,20)	1 (0,4)	1 (0,20)
Number of skin conditions identified on body map	3 (1,15)	4 (1,9)	3 (1,15)	2 (1,12)	2 (1,5)	2 (1,12)

### Molecular epidemiology of GAS isolates isolated from pharyngeal or impetigo swabs

**Fig 4:**
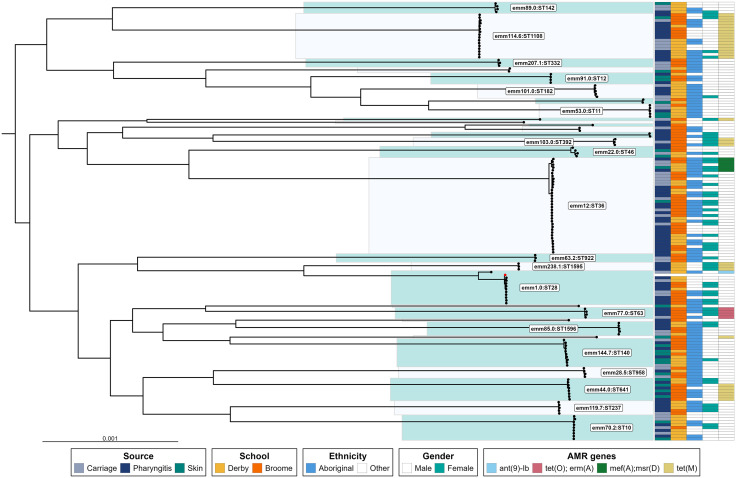
Phylogenetic tree of GAS isolates from each positive throat and skin swab specimen. The midpoint rooted, core-genome SNP phylogeny was generated using Gubbins and Snippy as described in the text. Branch lengths are given in substitutions per site as represented by the scale bar. Highlighted clades represent single unique emm-ST combinations. Coloured bars indicate isolate metadata including school setting, swab source (carriage, pharyngitis and skin), ethnicity, gender and known AMR genes.

The full list of sequenced genomes is available in S1 Data. Two-hundred and sixty-seven GAS isolates were cultured, of which 159 were whole genome sequenced (where possible, one GAS isolate per positive swab was sequenced). The majority (132/159, 83%) were derived from throat swabs, with 27/159 (17%) derived from skin. Genome sequencing of all GAS isolates identified 29 unique *emm* types that each correlated with evolutionary distinct MLST profiles (Fig 4). Strains within *emm* types consistently shared an MLST genotype. Known antimicrobial resistance determinants (including those encoding tetracycline, erythromycin, macrolide, and aminoglycoside resistance) were detected but their presence was not consistent or, detected in just 25.8% (41/159 isolates) of all strains. *emm*-types appeared to be distributed across all demographic subgroups including ethnicity, gender and age, with some minor *emm*-types (26 isolates from 12 *emm* types) exclusively found in either school. All *emm* types represented by at least three isolates were found in more than one niche (skin, carriage, pharyngitis).

There was a shift in the distribution of the most common *emm*-types over the study period. The frequency of swab collection was greater in the pre-COVID period (519 swabs in one year) versus the post-COVID period (516 swabs in ~18 months). The distribution of *emm*-types changed over the study period, with *emm12.0* (27/83 isolates), *emm1.0* (9/83 isolates), and *emm44.0* (8/83 isolates) being most common during the pre-COVID period (2019); and *emm114.6* (14/45 isolates) and *emm114.7* (11/45 isolates) being most common post-covid (2021–2022). The globally prevalent *emm12* genotype was prevalent during the pre-COVID period and remained in circulation post-COVID but was less frequently detected with time. Other *emm* types were detected sporadically (less than 10% of all isolates) throughout the surveillance period (*emm4.0, emm22.0, emm28.5, emm41.2, emm44.0, emm52.1, emm53.0, emm63.2, emm70.0, emm75.0, emm 77.0, emm81.0, emm82.0, emm82.1, emm85.0, emm89.0, emm91.0, emm101.0, emm103.0, emm104.0, emm105.0, emm119.7, emm207.1, emm238.1*).

A primary question of this study was to determine whether concurrent skin and throat infections were caused by the same *emm*-type simultaneously within individuals. We determined 12 instances of concurrent GAS impetigo with GAS pharyngitis/carriage in the same participant (11 of 254 individuals), each with unique circumstances. Of the 12 concurrent instances, the same *emm-*type was detected in each the throat and skin swab on nine occasions (75%). Isolates derived from instances of same *emm-types r*epresented 35% (9/26) of skin swabs collected during the study.

Twenty-two individuals experienced repeated detection of the same *emm* type. This included two individuals with repeat isolations of the same strain from skin (*emm12.0, emm53.0*), one individual with repeat isolation of the same strain from throat carriage only (*emm12.0*), and 19 individuals with repeat isolation of the same strain in the throat with at least one occurrence of pharyngitis (*emm 1.0, emm44.0, emm77.0, emm12.0, emm101.0, emm 114.6, emm114.7, emm238.1*).

### Detection of anti-streptococcal antibodies

A total of 379 DBS provided sufficient material for ASOT measurement. When comparing together (regardless of visit type), ASOTs ranged from 3 to 1834 IU/mL (median 199.9, IQR: 96.8–383.9). Thirty two percent of samples were above the upper limit defined as normal for the age range 5–14 years [32].

**Fig 5:**
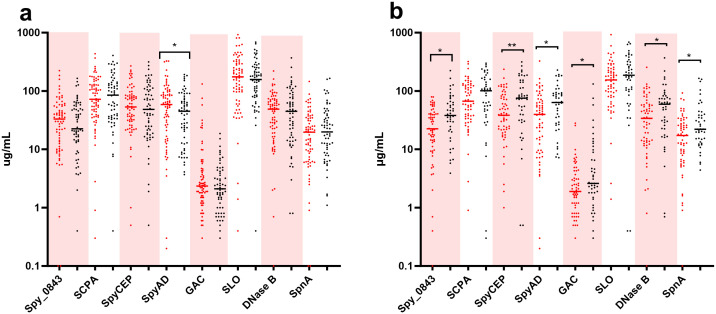
Detection of anti-streptococcal antibodies (µg/mL) with an 8-plex immunoassay. Available blood samples underwent analysis for antibodies targeting antigens used in clinical serology for ARF diagnosis (Streptolysin O = SLO, Deoxyribose nuclease B = DNase B and S. pyogenes nuclease A = SpnA) and five candidate vaccine antigens (Leucine-rich repeat domain-containing protein = Spy_0848, Streptococcal C5a peptidase = SCPA, S. pyogenes cell envelope protease = SpyCEP, S. pyogenes adhesion and division protein = SpyAD, and the group A carbohydrate = GAC). Where multiple samples were available for an individual the latest in date has been used for between-group comparisons. A: Comparison between Aboriginal participants (red circles, n = 53) and non-Aboriginal participants (black circles, n = 52). B: Comparison between participants without (red circles, n = 63) and with (black circles, n = 45) at least one GAS infection recorded. P-values from application of a Mann Whitney test are indicated as * 0.01 < p < 0.05 and **p < 0.01.

ASOT mean and medians were not different comparing prospective (n = 80) and active screening (n = 298) samples (p = 0.45). 8-plex immunoassay data was available for 137 DBS samples (Fig 5). Of the eight antigens tested, the most elevated titre was to SLO and the lowest titres were antibodies binding GAC. Differences in mean titre and range when comparing samples from Aboriginal (n = 53) and non-Aboriginal participants (n = 52) were small (Fig 5A). Children who observed at least one GAS infection (vs no GAS infection) had significantly higher antibody titres against Spy_0843, SpyCEP, SpyAD, GAC, DnaseB and SpnA but not to SCPA or SLO (Fig 5B). Antibody titres within individuals changed between sampling timepoints (including pre- and post- infection), but there was no consistent rise or decline, across all antigens. There was no appreciable rise in antibody titres with increasing age (S3 Fig).

## Discussion

This study enabled comprehensive surveillance of superficial GAS infections in school children living in a region at increased prevalence of ARF and RHD. Our primary aim was to determine the epidemiology of GAS-positive infections, particularly pharyngitis, through performing school-based prospective surveillance. We found a 6.3% (95% CI: 3.8%–10.6%) point prevalence of GAS-positive pharyngitis at screening visits. Comparison with other similar studies is hindered by the fact that our GAS-positive pharyngitis definition did not include serology, partly as it was not considered feasible to obtain post-infection (convalescent blood); the large rates of refusal to assent to finger pricking for blood collection (~63%) verified this prediction. However, GAS infections do not always elicit consistent serological immune responses [33] and limiting our diagnoses to positive serology would underreport the true GAS burden. We confirm that GAS-positive pharyngitis is more common among children at school in the Kimberley region than has been reported for this age group elsewhere in Australia (including no GAS pharyngitis reported in a survey of 2,695 children aged <15 residing in 3 remote Aboriginal communities) [10,12].

GAS pharyngitis represents a potentially significant reservoir for GAS transmission to classmates and/or family members, and places them at risk of ARF. However, detection of pharyngeal GAS carriage was also an important aim of this study, particularly in the context of recent studies describing asymptomatic carriage as key transmission drivers [8,9]. We found a higher rate of pharyngeal GAS carriage (9.0%, 95% CI: 7.5–10.7%) compared with GAS-positive pharyngitis. Of the swabs positive for GAS, 20% were collected from children with a truly asymptomatic presentation, with the remainder recording at least one sign or symptom of sore throat. A previous meta-analysis showed that pharyngeal GAS carriage prevalence is expected to be higher in high-income settings (8.4–12.9%) compared with low-income settings (4.3–8.1%), but the prevalence that we observed most closely resembles that of the high-income range [34]. A GAS-detection rate of approximately 15% of all pharyngeal swabs (combined symptomatic and asymptomatic burden) reinforces the need for accessible detection methods in remote-living Australians at risk of ARF, such as molecular point of care testing (POCT) [14,35]. The present study reports GAS identification with culture confirmation only, which is less sensitive and likely underestimates the total number of GAS positive swabs [36]. We describe elsewhere the detection of GAS using molecular POCT in this cohort (manuscript currently under review).

Aboriginal children are at increased risk of ARF and RHD and in this study we observed these children were more likely to have GAS pharyngitis, carriage and impetigo. However, GAS impetigo was the presentation with largest prevalence difference when comparing to non-Aboriginal children (PR 3.4, Table 3). This correlates with other settings where first nations children experience higher rates of ARF and RHD, specifically Māori and Pacific children, who have strikingly more GAS skin infections, but similar GAS pharyngitis rates compared to other children [5,37]. With small numbers of GAS impetigo, and no reported ARF and RHD in this cohort to date, we are unable to investigate this difference in relation to subsequent complications, but it may explain some of the increased risk in Aboriginal populations and should be further explored in larger studies.

We designed surveillance methodology to concurrently detect GAS-positive impetigo, with the aim of establishing co-occurrence with GAS pharyngitis. Overall, GAS-impetigo was uncommon in this study (2.6%, 95% CI: 1.6–4.2%) compared to studies in the Kimberley (up to 50% prevalence has been reported) [38]. Of note was the higher rate of impetigo reported during prospective (all enrolled participants) vs. active surveillance (self-reporting participants) which highlights the potential for normalisation of this presentation amongst children, a phenomena previously identified in this region [39,40]. This potentially explains the lower impetigo rate observed in this study, and coincides with additional factors, i.e., the participating schools were in two large towns which have better household maintenance and infrastructure than what is found in the more remote areas of the Kimberley [7,38,41]. The lowest impetigo prevalence was detected in the post-COVID disruption era (Table 3). We did not record data on non-pharmaceutical interventions implemented in study schools in this era; however, following the implementation of regional lockdowns intended to suppress the spread of COVID-19 among remote-living populations, Western Australian schools were required to promote COVID-safe behaviours among students such as encouraging staying home when unwell, practicing good hygiene, maintaining distance from adults, and enabling air ventilation [42]. Previous research from Pakistan suggests handwashing practices reduce rates of impetigo [43]. It is possible that increased exposure of students to health hygiene messaging intended to prevent SARS-COV2 infection also contributed to the reduced detection of skin sores during this period. The use of additional molecular methods for the detection of GAS in skin swabs (e.g., qPCR) is likely to enhance the detection rate for GAS [44], and will be further explored in subsequent studies.

To inform future school and/or community guidelines for sore throat recognition and management, we aimed to identify the most common signs and symptoms associated with GAS pharyngitis. Sore throat and difficulty swallowing were the most common child-reported symptoms. However, we confirmed a wide variety of symptoms associated with GAS-positive pharyngitis, and did not find exclusive or typical symptom combinations. This suggests all signs and symptoms of sore throat should be included in health messaging. Tonsillar erythema and tonsillar swelling were the most important clinically identified signs and should be assessed for in clinical interactions. When we retrospectively applied clinical decision rules (Centor scores) to screening assessment data, we found that they did not effectively identify pharyngitis cases: fewer than 50% of GAS pharyngitis cases were assigned a Centor score above 3 indicating higher chance of GAS-positive pharyngitis. It is possible that the application of clinical decision rules to this cohort is inappropriate, as children attending school are likely to be less unwell than those seeking medical attention. This possibility is supported by the very low detection of fever observed among participants. Similar findings have been reported in systematic reviews of clinical decision rules globally [45].

We aimed to sequence GAS strains from skin and throat infections to provide new insight into GAS strain distribution across niches within and between individuals. Overall, we found that *emm* types were diverse (29 unique *emm* types) but largely consistent across niches (skin vs. pharyngitis vs. pharyngeal carriage), communities, genders and ethnicities, to the exclusion of rare types. Globally dominant types (e.g., *emm12.0,* 21.6% of isolates in this study) remained persistent in the pre- and post-COVID-19 periods but fluctuated from being the dominant types to less common types over time. This contrasts with observations made in high-income settings; for example, a school-based surveillance study conducted in Pittsburgh identified fewer *emm* types in a larger GAS collection during a 4-year surveillance period [46]. However, our findings are similar to a more recent geographically relevant study of isolates from New Zealand children showing high overall diversity (49 *emm* types from 469 isolates) with maintenance of globally dominant strains [47]. Concurrent skin and throat infections (pharyngitis or pharyngeal carriage) were mostly (75%) but not always, due to the same *emm* type, suggesting the movement of infecting strains from between infection sites within an individual and demonstrated previously [8]. We identified several instances of a repeat *emm* type (pharyngitis or pharyngeal carriage) detection in individuals over long periods (e.g., > 1 year), a longer duration than previously reported [46]. We could not discern re-infection from long-term persistence, nor could we investigate whether these individuals received antibiotics, but long term detection has potential implications for ARF prevention in individuals, and potentially presents a considerable transmission risk to others. This also highlights the importance of reinforcing strategies that target both pharyngitis and impetigo in the context of ARF and RHD prevention in Australia. Our molecular epidemiological findings were largely consistent with the only other epidemiological study in Australia that has been performed in communities at risk of ARF [48].

Finally, we aimed to survey streptococcal antibody titres to identify correlations between infection, age and ethnicity and provide contemporary region-specific data for informing future vaccine implementation. Overall, antibody responses did not differ between ethnicities, despite Aboriginal children experiencing higher prevalences of pharyngeal GAS carriage and GAS-positive impetigo (prevalence ratio of 1.76 and 3.45, respectively Table 3). This contrasts with studies in New Zealand showing elevated average titres in healthy children of Indigenous Māori and Pacific ethnicity, populations with increased risk of ARF [49]. Anti-streptolysin O titres were detected above the upper limit of normal in approximately one third of the children tested in our study (when including active surveillance and prospective seasonal visits), and the elevated mean titres observed confirm the difficulties of using antibody testing to infer a recent GAS infection in a high burden setting. The relatively flat association between antibody titre and increasing age suggests that children have experienced several GAS infections before the age of 5 years and do not experience significant titre increases with additional infections in the age group studied here, something that has also been observed in a household-based study in the Gambia [50]. As GAS vaccines accelerate towards licensure, this is an important consideration when deciding ‘when’ and ‘who’ to vaccinate in Australia.

There are limitations to our study. Firstly, our study was performed in two private (fee-accepting) schools, with participants potentially experiencing higher socioeconomic circumstances compared with their age-matched peers. Despite this, ARF and RHD were diagnosed in both school communities during the study (none were study participants), confirming that these are important populations for surveillance. Secondly, our study spanned a significant pandemic-interruption period, which necessitates caution in the interpretation of overall rates. Thirdly, our school-based surveillance study did not enrol every student in the school community, and it may have missed the most severe cases of sore throats and skin sores given that students with these infections are advised to stay home from school – and were likely to be absent when active-surveillance occurred. Fourthly, our reliance on single time-point assessments during prospective screening and weekly active surveillance visits may have missed infections occurring outside school visits or in children who were absent. Additionally, children with unreported symptoms may not have met our case definition at the time of active screening, potentially leading to misclassification of true GAS pharyngitis cases. Overall, our findings are likely to be an under-estimation of the true burden of GAS in the two Kimberley school communities. Finally, we relied upon self-reported data describing the use of antibiotics alongside clinic audits, which did not identify a significant usage amongst enrolled children, limiting our ability to infer the impact of antibiotic treatment.

## Conclusion

In this 3-year longitudinal study, we detected GAS infections in a unique setting utilising contemporary surveillance methodologies. Our data describe GAS positivity rates in school-aged children in Australia, informing: infection prevalence in two communities over time, connections between concurrent skin and throat infections, and details of streptococcal immune responses in a high burden setting. The results of our study will serve further development of primary prevention activities such as changes to public health policy, community-controlled prevention and advocacy in Australia, the implementation of rapid GAS testing [35], and global vaccine development and strategy.

## Supporting information

S1 Fig Map of Western Australia showing the Kimberley region, Broome and Derby. Shapefiles for the map were retrieved from the Australian Bureau of Statistics Geocentric Datum of Australia 2020 (GDA2020) (available at: https://www.abs.gov.au/statistics/standards/australian-statistical-geography).
(TIFF)

S2 Fig Clinical ASOT via nephelometry correlates with 8-plex immunoassay. (TIF)

S3 Fig Antibody titres were stable with increasing child age. (TIF)

S1 Data Accession numbers for all GAS genomes used in phylogenetic analysis. (XLSX)
