# Supplementary figures and images for: Longitudinal surveillance of group A streptococcal pharyngitis and impetigo in remote Western Australian school children informs acute rheumatic fever prevention
Source: PLOS Glob Public Health. 2025 Dec 19;5(12):e0005398. doi: 10.1371/journal.pgph.0005398 (PMC12716692; doi:10.1371/journal.pgph.0005398)

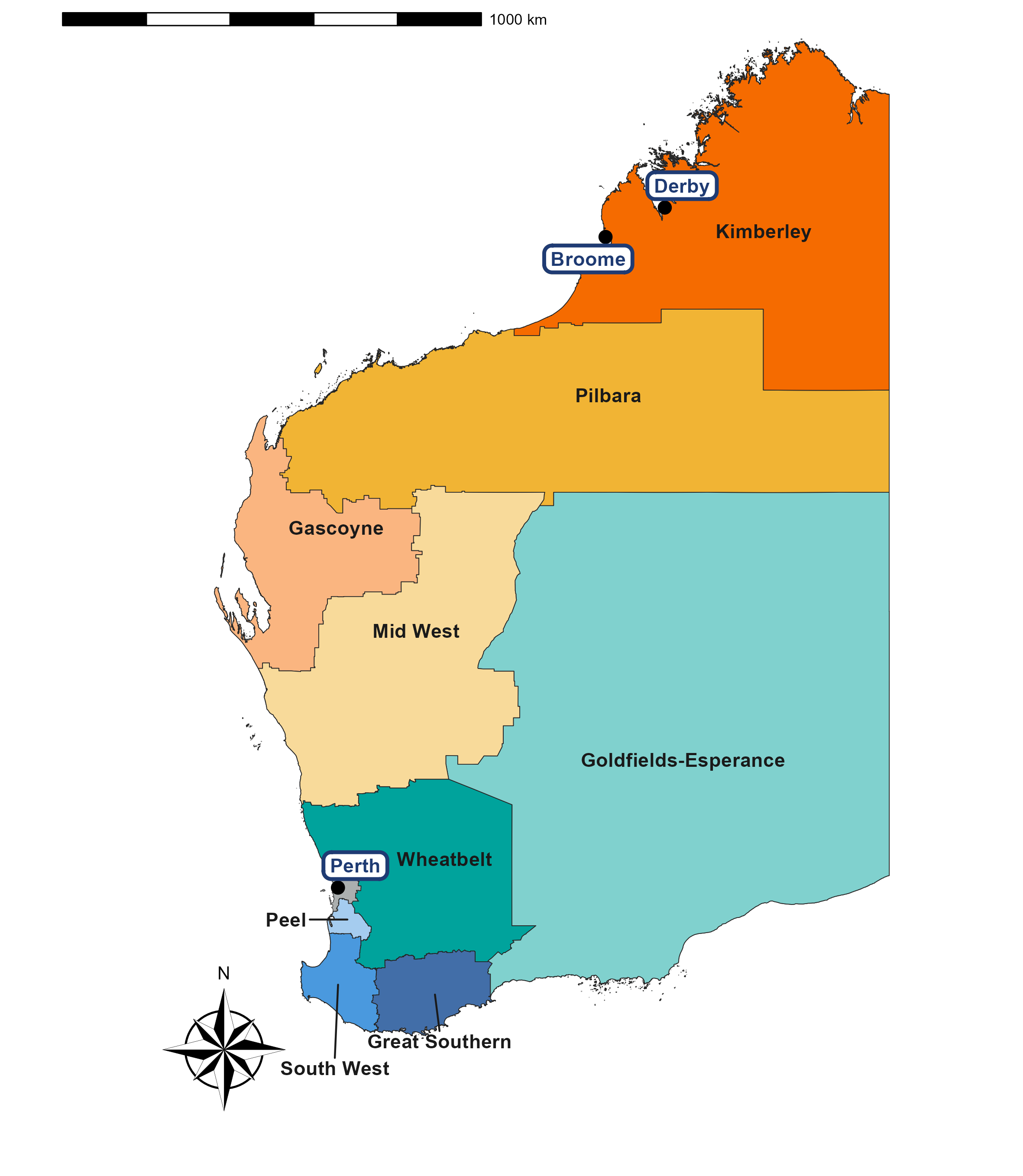

Supplement: S1 Fig — Shapefiles for the map were retrieved from the Australian Bureau of Statistics Geocentric Datum of Australia 2020 (GDA2020) (available at: https://www.abs.gov.au/statistics/standards/australian-statistical-geography). (TIFF) [file pgph.0005398.s001.tiff]

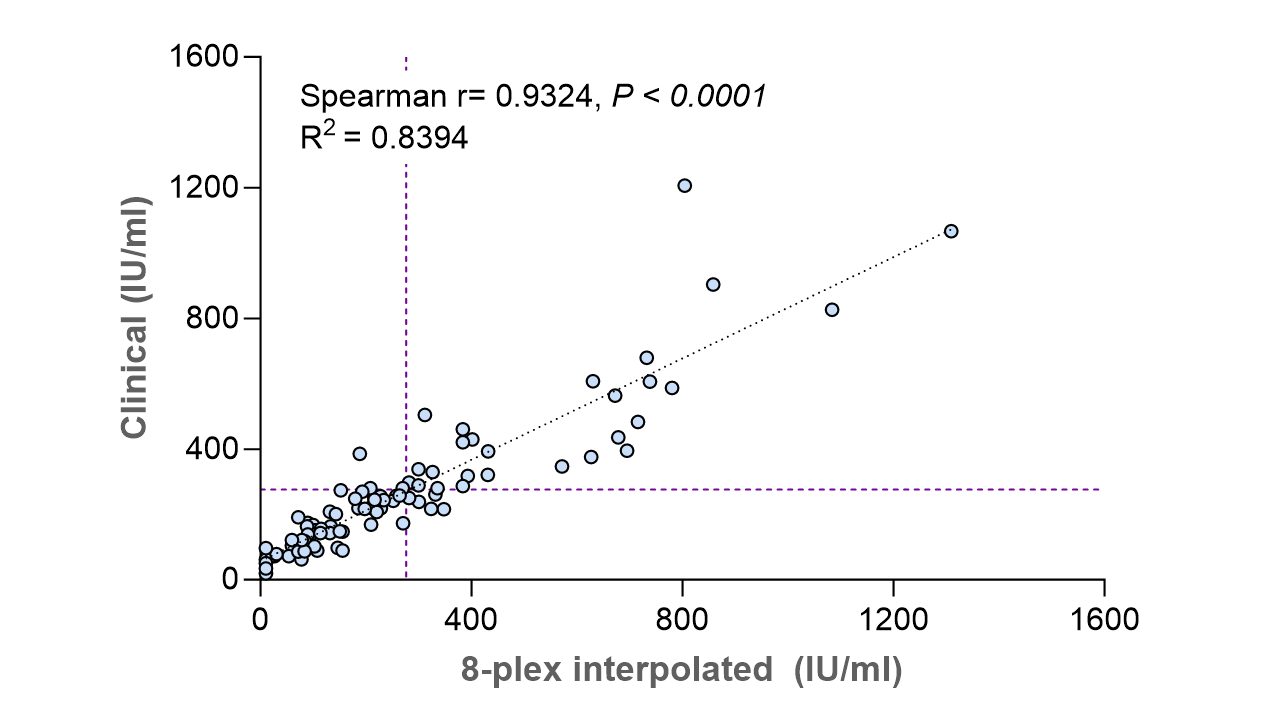

Supplement: S2 Fig — (TIF) [file pgph.0005398.s002.tif]

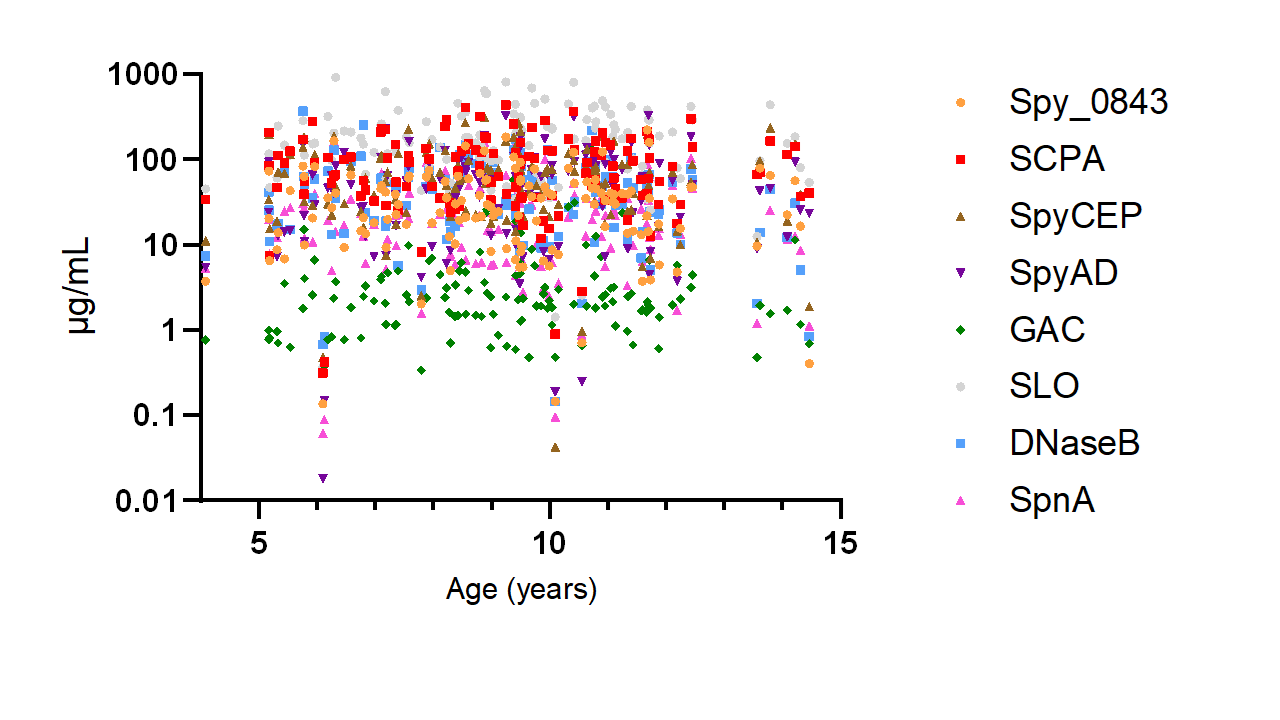

Supplement: S3 Fig — (TIF) [file pgph.0005398.s003.tif]
